# Maternal Genetic Variants and Gestational Duration: A Replication Study in a Japanese Cohort

**DOI:** 10.3390/jcm15114269

**Published:** 2026-06-01

**Authors:** Rina Tanabu, Kaori Iino, Maki Sato, Mako Nakamura, Macthi Yokoyama, Yoshinori Tamada, Ken Itoh, Tatsuya Mikami, Koichi Murashita, Yoshihito Yokoyama

**Affiliations:** 1 Department of Obstetrics and Gynecology, Graduate School of Medicine, Hirosaki University, Hirosaki 036-8562, Japan; 2 Research Center for Health-Medical Data Science, Graduate School of Medicine, Hirosaki University, Hirosaki 036-8562, Japan; 3 Department of Stress Response Science, Biomedical Research Center, Graduate School of Medicine, Hirosaki University, Hirosaki 036-8562, Japan; 4 Department of Preemptive Medicine, Innovation Center for Health Promotion, Graduate School of Medicine, Hirosaki University, Hirosaki 036-8562, Japan; 5 Department of Research Institute of Health Innovation, Hirosaki University, Hirosaki 036-8224, Japan

**Keywords:** genome-wide association study, gestational duration, maternal genetics, preterm birth, single nucleotide polymorphism

## Abstract

**Background/Objectives**: The objective of this study is to evaluate whether maternal genetic variants previously associated with gestational duration in European-ancestry populations are associated with gestational duration in Japanese women. **Methods**: We analyzed 347 women with a history of delivery from a community-based cohort in Aomori Prefecture, Japan. Gestational age at first delivery (weeks) was obtained from the Maternal and Child Health Handbook. Four representative maternal single-nucleotide polymorphisms were analyzed: rs2946169 near *EBF1*, rs2955117 in/near *EEFSEC*, rs12037376 in/near *WNT4*, and rs9861425 in/near *ADCY5*. Maternal genotype data were obtained from existing Japonica Array data generated as part of the Iwaki Health Promotion Project. Associations with gestational age at first delivery were tested using linear regression under an additive genetic model, adjusted for maternal age at first delivery. A Bonferroni-corrected threshold of *p* < 0.0125 was applied for four SNPs. **Results**: Mean gestational age at first delivery was 39.4 weeks. Each additional rs2946169 T allele was nominally associated with shorter gestation (adjusted β = −0.2679 weeks per allele; *p* = 0.021), but this association did not remain significant after Bonferroni correction. No significant associations were observed for rs2955117, rs12037376, or rs9861425. **Conclusions**: These findings provide suggestive evidence that maternal variation at the *EBF1* locus may be related to gestational duration in Japanese women. However, the association did not remain significant after multiple-testing correction, and larger studies are needed to confirm this finding and clarify population-specific genetic effects.

## 1. Introduction

The timing of delivery is a critical determinant of neonatal outcomes. Spontaneous preterm birth (delivery before 37 completed weeks of gestation) is a leading direct cause of neonatal mortality and remains a major contributor to under-five mortality worldwide [1]. An estimated 15 million infants are born preterm each year, making preterm birth a major public health challenge [2]. Conversely, excessively prolonged gestation (post-term pregnancy, ≥42 weeks) is associated with increased fetal and maternal risks, including a marked rise in stillbirth rates particularly after 41–42 weeks [3]. Maintaining gestational duration within an appropriate range is therefore of paramount importance in perinatal care.

Gestational duration is influenced by a complex interplay of maternal, fetal, and environmental factors. Established risk factors for shortened gestation include extreme maternal age, smoking, infection, malnutrition, and adverse socioeconomic conditions. However, these factors alone do not sufficiently explain the substantial inter-individual variation in gestational length. Family and twin studies indicate that approximately 30–40% of the variability in gestational duration and preterm birth risk is attributable to genetic factors [4,5]. Moreover, this genetic contribution is thought to derive predominantly (though not exclusively) from the maternal genome, suggesting that maternal genes involved in the maintenance of pregnancy play an important role in determining the timing of labor onset [5].

In recent years, genomic studies have identified maternal loci associated with gestational duration and spontaneous preterm birth [6,7,8,9]. Large-scale genome-wide association studies (GWAS) in populations of European ancestry have reported maternal variants at genes such as *EBF1*, *EEFSEC*, *WNT4*, *ADCY5*, *AGTR2*, and *RAP2C* to be associated with gestational length [6,9]. Some of these loci (e.g., *EBF1*, *EEFSEC*, and *AGTR2*) have also been linked to preterm birth risk. The implicated genes participate in biological processes relevant to the timing of parturition, including uterine development, hormonal signaling, maternal nutrition, and vascular regulation, thereby improving our understanding of the mechanisms underlying pregnancy maintenance and the initiation of labor [7,8]. However, most genetic discoveries to date are heavily skewed toward populations of European ancestry, and it remains unclear whether these findings generalize to other ethnic groups. Differences in allele frequencies, linkage disequilibrium structure, and baseline preterm birth rates may lead to population-specific genetic effects [7,8]. For example, Japan has a lower prevalence of spontaneous preterm birth than Western countries [10]. Replication studies in non-European populations are therefore essential to determine the transferability of these genetic risk factors [7,8,11].

The aim of this study was to assess whether maternal genetic polymorphisms associated with gestational duration in European-ancestry populations are also valid in a Japanese population. Among previously reported loci associated with gestational duration and/or spontaneous preterm birth, nine SNPs were available in the Japonica Array dataset used in the present cohort. From these available SNPs, we selected four representative maternal SNPs for the primary analysis: rs2946169 near *EBF1*, rs2955117 in/near *EEFSEC*, rs12037376 in/near *WNT4*, and rs9861425 in/near *ADCY5*. By testing these associations in a Japanese cohort, this study aimed to provide evidence regarding the cross-population transferability of genetic factors influencing gestational duration.

## 2. Materials and Methods

### 2.1. Study Design and Participants

**Figure 1:**
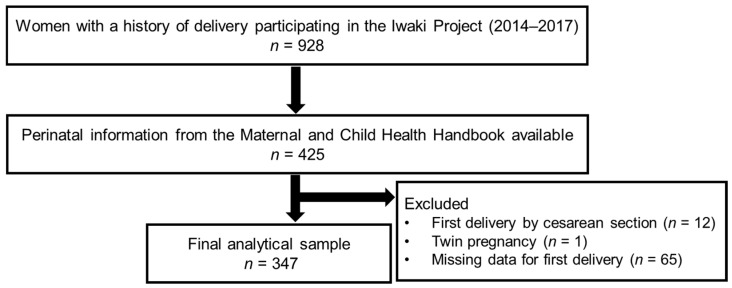
Flow diagram of participant selection. Women with a history of delivery participating in the Iwaki Project (2014–2017) were identified (*n* = 928). Perinatal information from the Maternal and Child Health Handbook was available for 425 women. After excluding women whose first delivery was by cesarean section (*n* = 12), twin pregnancy (*n* = 1), and those missing data for their first delivery (*n* = 65), 347 women were included in the final analysis.

This study was conducted as part of the Iwaki Health Promotion Project, an ongoing community-based cohort study in Hirosaki City, Aomori Prefecture, Japan [12]. Women with a history of delivery who participated in the project between 2014 and 2017 were eligible for the current analysis. Participants were recruited from a geographically defined community in Aomori Prefecture and were presumed to be predominantly of Japanese ancestry based on cohort eligibility and residence. The participant selection flow diagram is shown in Figure 1. The inclusion criteria were as follows: participation in the Iwaki Health Promotion Project between 2014 and 2017, a history of delivery, available perinatal information from the Maternal and Child Health Handbook, and available genotype data for at least one of the candidate SNPs. Among 928 women with a history of delivery in the cohort, 425 had available perinatal information recorded in the Maternal and Child Health Handbook. We further excluded women whose first delivery was by cesarean section (*n* = 12) or twin pregnancy (*n* = 1) to focus on singleton first deliveries, as well as those missing data for their first childbirth (*n* = 65). These exclusions were made to minimize the influence of obstetric intervention, such as cesarean delivery, and multiple gestation on gestational length and to better reflect the natural course of gestational duration in singleton first pregnancies. The final analytic sample comprised 347 women (Figure 1). Information on pregnancy complications, such as preeclampsia and gestational diabetes, and chronic maternal diseases at the time of first pregnancy was not systematically available for all participants from the available records. Therefore, these conditions could not be included as exclusion criteria or covariates in the main analysis.

Written informed consent was obtained from all participants, and the study protocol was approved by the Ethics Committee of Hirosaki University School of Medicine (Approval code; 2014-014, 2014-377-1, 2016-028-1, 2021-030).

Gestational age at first delivery (weeks) was obtained from the Maternal and Child Health Handbook records. In Japan, all pregnant women receive a Maternal and Child Health Handbook—a standardized record in which obstetric healthcare providers document medical history, maternal characteristics, antenatal measurements, and clinical findings throughout pregnancy. For this analysis, we used each woman’s first delivery to standardize parity. Birth outcomes, including birth weight, were also collected from the same records. Maternal age at first delivery (years), used as an adjustment variable in the regression models, was also obtained from the Handbook records and summarized in Table 1. The relatively high mean age at study enrollment reflects the retrospective nature of this analysis, in which historical first-delivery information was obtained from women participating in a community-based health examination. The age relevant to the pregnancy analyzed in this study was maternal age at first delivery. Because many women retain their Handbook for decades, these records provide a reliable source of historical perinatal information for epidemiologic studies [13,14,15].

### 2.2. Genotyping and SNP Selection

Maternal genotype data were obtained from genome-wide genotype data previously generated as part of the Iwaki Health Promotion Project. The present study did not perform new DNA extraction or new genotyping; instead, we used existing Japonica Array genotype data available in the Iwaki Project dataset.

Genotypes were determined using a genome-wide SNP array optimized for Japanese populations, the Japonica Array, which was developed by the Tohoku Medical Megabank Organization, Tohoku University, Sendai, Japan [16]. The Japonica Array was designed based on whole-genome sequencing data from Japanese individuals and is suitable for population-based genetic studies in Japanese cohorts. Whole-genome sequencing data were also available for a subset of participants to validate genotyping accuracy. The genotype data used in the present analysis had undergone standard quality control procedures in the Iwaki Project/Japonica Array dataset, including checks for genotype call rate and Hardy–Weinberg equilibrium. Concordance was verified using available whole-genome sequencing data in a subset of participants. Because this study used existing quality-controlled genotype data, no additional laboratory DNA extraction or genotyping procedures were performed specifically for the present analysis.

Among SNPs located in previously reported loci associated with gestational duration and/or spontaneous preterm birth, nine SNPs were available in the Japonica Array dataset used in the Iwaki cohort. When two SNPs were available for the same gene or genomic region, one representative SNP was selected for the primary analysis to avoid redundant testing of closely related markers. Selection was based on similarity of association results, interpretability of variant annotation, suitability for additive allele coding, genotype data availability, and allele frequency in the Japanese/Iwaki cohort. The full list of available SNPs and the rationale for representative SNP selection are shown in Supplementary Table S1.

The four representative SNPs selected for the primary analysis were rs2946169 near *EBF1*, rs2955117 in/near *EEFSEC*, rs12037376 in/near *WNT4*, and rs9861425 in/near *ADCY5*. Other loci reported in previous GWAS, including AGTR2 and RAP2C, were not included in the primary analysis because suitable variants were not available in the Japonica Array dataset or did not meet the predefined criteria for representative SNP selection.

Due to missing genotype data for some SNPs, the number of non-missing observations was 347 for rs2946169 and rs9861425, 338 for rs2955117, and 330 for rs12037376. Genotype distributions and minor allele frequencies (MAF) for each SNP are shown in Table 2.

Minor alleles were defined according to the previously published GWAS of gestational duration [6]. Because allele frequencies differ across populations, the minor allele in this Japanese sample did not necessarily correspond to the effect allele reported in European-ancestry populations. Genotypes were coded as 0, 1, or 2 copies of the GWAS-defined minor allele. MAF was calculated as the frequency of the GWAS-defined minor allele from observed genotype counts in this analytical sample. For reference, allele frequencies in other populations (1000 Genomes Project super-populations) are provided in Table 3 [17].

### 2.3. Statistical Analysis

Associations between each representative SNP and gestational age at first delivery were evaluated using linear regression. SNPs were modeled under an additive genetic model, coded as 0, 1, or 2 according to the number of minor alleles, and the outcome was gestational age at first delivery in weeks. Each SNP was analyzed separately in an individual regression model. We first fitted unadjusted models for each SNP, and then fitted multivariable models adjusted for maternal age at first delivery (years). The regression coefficient β represents the change in gestational age (weeks) per additional copy of the minor allele.

Nominal statistical significance was defined as a two-sided *p* < 0.05. To account for testing of four representative SNPs, a Bonferroni-corrected significance threshold of *p* < 0.0125 was also applied. Results with *p* < 0.05 but not meeting the Bonferroni-corrected threshold were interpreted as nominal associations.

In addition to the primary additive genetic model, dominant and recessive genetic models were examined as sensitivity analyses using age-adjusted linear regression models. In the dominant model, heterozygous and minor-allele homozygous genotypes were combined and compared with the major-allele homozygous genotype. In the recessive model, the minor-allele homozygous genotype was compared with the combined group of heterozygous and major-allele homozygous genotypes.

Exploratory logistic regression analyses were also performed using preterm birth, defined as delivery before 37 completed weeks of gestation, as a binary outcome. SNPs were coded as 0, 1, or 2 according to the number of minor alleles and analyzed under an additive genetic model. These models were adjusted for maternal age at first delivery. Because the number of preterm births was small, the logistic regression results were considered exploratory.

Post hoc power analysis was performed for the age-adjusted additive linear regression models. For each SNP, power was estimated using the observed regression coefficient, standard error, and residual degrees of freedom from the corresponding model. We calculated the power to detect the observed effect size at α = 0.05 and at the Bonferroni-corrected threshold of α = 0.0125. We also estimated the minimum detectable effect size with 80% power at both significance thresholds.

Given the geographically restricted, community-based cohort and the candidate-variant replication design, we did not additionally adjust for population stratification using ancestry principal components. Although the cohort consisted of individuals from a geographically and ethnically homogeneous Japanese population, subtle population structure within Japan cannot be completely excluded. Therefore, residual confounding due to unmeasured population stratification remains possible and should be considered when interpreting the findings.

All analyses were performed using R software, version 4.0.5 (R Foundation for Statistical Computing, Vienna, Austria). EZR, version X.XX (Saitama Medical Center, Jichi Medical University, Saitama, Japan), a graphical user interface for R, was used for selected statistical analyses [18].

## 3. Results

### 3.1. Participant Characteristics

A total of 347 women were included in the analysis. The mean age at study enrollment was 50.8 ± 13.0 years. The mean gestational age at first delivery was 39.4 ± 1.4 weeks. Key participant characteristics are summarized in Table 1. Preterm birth, defined as delivery before 37 completed weeks of gestation, occurred in 9 women (2.6%). Low birth weight (<2500 g) was observed in 18 infants (5.2%). Among singleton births, the mean birth weight was 3115.2 ± 370.5 g.

### 3.2. Associations Between Maternal SNPs and Gestational Duration

We evaluated the association of each representative maternal SNP with gestational age at first delivery in weeks. Table 4 presents the linear regression results for each SNP in both unadjusted models and models adjusted for maternal age at first delivery. In the unadjusted analysis, rs2946169 near *EBF1* showed a nominal association with shorter gestational age at first delivery. Each additional copy of the minor T allele of rs2946169 was associated with a shorter gestational duration (unadjusted β = −0.2663 weeks, 95% CI: −0.4941 to −0.0384, *p* = 0.022; *n* = 347). This association remained nominally significant after adjustment for maternal age at first delivery (adjusted β = −0.2679 weeks, 95% CI: −0.4948 to −0.0410, *p* = 0.021). However, this result did not meet the Bonferroni-corrected significance threshold of *p* < 0.0125.

The effect size corresponded to approximately −0.27 weeks, or about 1.9 days, per additional T allele. No significant associations were observed for rs2955117, rs12037376, or rs9861425 in either unadjusted or age-adjusted models. In the age-adjusted models, the effect estimates for these SNPs were close to zero, and all *p* values were >0.70.

Post hoc power analysis showed that the power to detect the observed effect of rs2946169 was 63.9% at α = 0.05 and 42.6% at the Bonferroni-corrected threshold of α = 0.0125 (Supplementary Table S2). The minimum detectable effect sizes with 80% power ranged from 0.310 to 0.460 weeks at α = 0.05 and from 0.371 to 0.550 weeks at α = 0.0125, indicating limited power to detect small genetic effects.

In exploratory logistic regression analyses using preterm birth as a binary outcome, none of the four representative SNPs showed a statistically significant association with preterm birth after adjustment for maternal age at first delivery (Supplementary Table S3).

Sensitivity analyses using dominant and recessive genetic models generally supported the primary additive model results for rs2946169, with a consistent direction of association toward shorter gestational duration (Supplementary Table S4). A nominal association was also observed for rs2955117 under the recessive model; however, this finding should be interpreted cautiously because no association was observed in the primary additive model.

## 4. Discussion

This study examined whether maternal genetic polymorphisms previously reported to be associated with gestational duration in populations of European ancestry are also applicable to a Japanese population. We observed a nominal association between rs2946169 near *EBF1* and shorter gestational duration in Japanese women, whereas no significant associations were observed for rs2955117, rs12037376, or rs9861425. However, the association for rs2946169 did not remain statistically significant after Bonferroni correction for four representative SNPs. Therefore, our findings should be interpreted as suggestive evidence rather than definitive replication. The direction of effect for rs2946169 was consistent with previous GWAS findings, suggesting that this locus may contribute to gestational duration across populations, although confirmation in larger Japanese and other East Asian cohorts is required.

*EBF1* (Early B-cell Factor 1) is a transcription factor best known for its role in B-cell differentiation; however, accumulating evidence suggests broader roles in pregnancy maintenance and parturition. A large GWAS identified rs2946169 as a genome-wide significant locus for gestational duration and reported that the T allele was associated with shorter gestation and an increased risk of spontaneous preterm birth [6]. In the present study, each additional T allele of rs2946169 was nominally associated with a 0.2679-week shorter gestational duration, corresponding to approximately 1.9 days. Although the direction of effect was consistent with prior reports, the effect size was modest and the association did not survive multiple-testing correction. Furthermore, exploratory logistic regression analysis did not show a significant association between rs2946169 and preterm birth. Therefore, this finding should be interpreted as a modest signal for gestational duration as a continuous trait, rather than evidence that rs2946169 alone predicts clinically meaningful risk of preterm birth. The relatively high minor allele frequency of rs2946169 across populations may also partly explain why this association could be detected even in a cohort of a few hundred participants.

In contrast, we did not observe significant associations for rs2955117, rs12037376, or rs9861425 in our Japanese sample. Several explanations should be considered. First, allele frequencies and linkage disequilibrium patterns differ across populations, which can substantially affect statistical power. Population-specific allele frequencies derived from the 1000 Genomes Project (Table 3) illustrate marked differences between East Asian and European populations, supporting this possibility [17]. Second, our limited sample size may have reduced power to detect very small genetic effects. The post hoc power analysis indicated that this study had 80% power to detect per-allele effects of approximately 0.310–0.460 weeks at α = 0.05 and 0.371–0.550 weeks at the Bonferroni-corrected threshold. These detectable effect sizes were larger than the observed estimates for rs2955117, rs12037376, and rs9861425. Therefore, the null findings for these SNPs should not be interpreted as evidence of no association, but rather as inconclusive findings in a modest-sized replication sample.

From a biological perspective, the lack of association in our study does not negate the plausibility of these genes in influencing gestational length. *WNT4* plays a critical role in female reproductive tract development and has been implicated in implantation and decidualization processes, which are essential for pregnancy maintenance [19]. It is possible that different variants in *WNT4* are relevant in East Asian populations, or that the effect sizes of the specific *WNT4* SNPs we examined are too small to detect in the present study. *EEFSEC* encodes a factor involved in selenocysteine incorporation, and previous GWAS have suggested its relevance to gestational duration and spontaneous preterm birth [6]. In the present study, rs2955117 showed no association under the primary additive model, although a nominal association was observed under the recessive model in sensitivity analysis. Because this recessive-model finding was not supported by the primary additive model and may be unstable due to the small number of minor-allele homozygotes, it should be interpreted cautiously. *ADCY5*, encoding adenylate cyclase 5, has been associated with fetal growth and birth weight in large genetic studies, primarily through fetal genetic effects [20,21]. These findings raise the possibility that maternal or fetal variation in *ADCY5* could influence pathways affecting gestational timing or fetal growth in ways not captured by our analysis of gestational duration alone.

Recent large-scale multi-ancestry meta-analyses have highlighted the importance of including diverse populations to identify novel loci and refine the genetic architecture of gestational duration [22]. Such studies demonstrate that some genetic effects are shared across ancestries, whereas others are population-specific [23]. Our findings are consistent with this broader framework in that rs2946169 showed a nominal association in the same direction as previous GWAS findings, while the other representative SNPs did not show significant associations in this Japanese cohort. These results underscore the need for larger Japanese GWAS and international collaborations to fully elucidate the genetic determinants of gestational length.

Several strengths of this study merit consideration. We utilized high-quality gestational age data derived from the Maternal and Child Health Handbook, which provides reliable, physician-recorded pregnancy information even many years after delivery. We also restricted the analysis to singleton first deliveries by excluding women whose first delivery was by cesarean section and multiple pregnancies, and we adjusted for maternal age at first delivery to account for its potential confounding effect. In addition, we used representative SNPs selected from available Japonica Array variants and provided the full list of available SNPs and selection rationale in Supplementary Table S1, improving transparency of the candidate-SNP selection process. Prior work has reported that maternal circulating *EBF1* mRNA levels differ in cases of spontaneous preterm birth, providing functional support for the role of this locus in parturition [24].

Limitations of this study should also be acknowledged. The sample size was relatively small, which limits our power to detect variants with very small effects. Only a small number of candidate SNPs were examined, and the cohort was drawn from a single geographic region in Japan, which may limit generalizability. Although rs2946169 showed a nominal association with gestational duration, it did not remain significant after Bonferroni correction. Therefore, the result should be interpreted cautiously. In addition, we did not adjust for population stratification using principal components derived from genome-wide genotype data. Because this study was designed as a candidate-variant replication analysis, subtle confounding due to population structure cannot be completely excluded. Information on pregnancy complications, such as preeclampsia and gestational diabetes, and chronic maternal diseases at the time of first pregnancy was not systematically available for all participants; therefore, these factors could not be included as exclusion criteria or covariates. The number of preterm births was also small, limiting our ability to evaluate genetic associations with preterm birth as a binary outcome. Moreover, we did not analyze some loci identified in the original GWAS, such as AGTR2 and RAP2C, because suitable variants were not available in the Japonica Array dataset or did not meet the predefined criteria for representative SNP selection. These factors should be considered when interpreting the absence of association for rs2955117, rs12037376, and rs9861425 in our results.

Future studies should integrate genetic factors with clinical and environmental predictors of gestational duration, such as prior preterm birth history, cervical length, infection-related biomarkers, maternal body mass index, smoking, hypertensive disorders of pregnancy, diabetes, nutritional status, socioeconomic conditions, and psychosocial stress. Gene–environment interactions may partly explain differences in genetic effect estimates across populations and should be evaluated in larger datasets. Although the effects of individual variants are modest, combining genetic markers with established risk factors may improve the prediction of abnormal delivery timing. Additionally, functional studies will be essential to clarify how *EBF1* and related molecular pathways contribute to the maintenance of pregnancy and the initiation of labor.

## 5. Conclusions

Maternal genetic variation at the *EBF1* locus, rs2946169, showed a nominal association with gestational duration in Japanese women, with the same direction of effect as previously reported in European-ancestry populations. However, this association did not remain statistically significant after Bonferroni correction, and exploratory logistic regression analysis did not show a significant association with preterm birth. In contrast, no significant associations were observed for rs2955117, rs12037376, or rs9861425, which may be attributable to differences in allele frequencies and linkage disequilibrium structure between populations and/or limited statistical power in this modest-sized cohort. These findings provide suggestive evidence that maternal variation near *EBF1* may be related to gestational duration in Japanese women, but they require confirmation in larger, adequately powered studies with comprehensive adjustment for ancestry, clinical factors, and environmental exposures. Further studies in diverse populations, together with functional characterization of *EBF1*-related pathways, are warranted to refine the genetic determinants of gestational timing and their relevance to adverse outcomes such as spontaneous preterm birth.

## Figures and Tables

**Table 1:** Participant characteristics (*n* = 347).

Characteristics at Enrollment	
Age (years)	50.8 ± 13.0
Body mass index (kg/m^2^)	22.2 ± 3.4
First birth characteristics	
Age at first delivery (years)	26.8 ± 4.9
Gestational age at delivery (weeks)	39.4 ± 1.4
Preterm birth, *n* (%)	9 (2.6%)
Low birth weight (<2500 g), *n* (%)	18 (5.2%)
Birthweight (g)	3115.2 ± 370.5
Infant sex (male/female), *n* (%)	194 (56.2%)/151 (43.8%)

Notes. Values are presented as mean ± standard deviation or *n* (%). Infant sex data were missing for two participants.

**Table 2:** Genotype distribution and minor allele frequency of the four analyzed SNPs in the Iwaki analytical sample.

SNP	N	Genotype Counts *n* (%)	Minor Allele (MAF%)
rs2946169	347	CC 185 (53.3%) TC 128 (36.9%) TT 34 (9.8%)	T (28.2%)
rs2955117	338	GG 251 (74.3%) AG 82 (24.3%) AA 5 (1.5%)	A (13.6%)
rs12037376	330	AA 93 (28.2%) GA 154 (46.7%) GG 83 (25.2%)	G (48.5%)
rs9861425	347	AA 128 (36.9%) CA 168 (48.4%) CC 51 (14.7%)	C (38.9%)

Notes. Sample size differs by SNP because of missing genotype data. Abbreviations: SNP, single-nucleotide polymorphism.

**Table 3:** Minor allele frequencies (MAF) of the analyzed SNPs in Japanese and other reference populations (1000 Genomes super-populations).

Gene	SNP	JPT	EAS	EUR	AFR
*EBF1*	rs2946169	0.27	0.27	0.20	0.48
*EEFSEC*	rs2955117	0.14	0.05	0.29	0.35
*WNT4*	rs12037376	0.57	0.45	0.16	0.04
*ADCY5*	rs9861425	0.36	0.25	0.46	0.71

Notes. JPT, Japanese in Tokyo; EAS, East Asian; EUR, European; AFR, African. Reference population allele frequencies were obtained from the 1000 Genomes Project (Phase 3).

**Table 4:** Unadjusted and age-adjusted linear regression results for associations between maternal SNPs and gestational age at first delivery (weeks).

SNP	Unadjusted β [95% CI]	Unadjusted *p*	Adjusted β [95% CI]	Adjusted *p*	N
rs2946169	−0.2663 [−0.4941, −0.0384]	0.022	−0.2679 [−0.4948, −0.0410]	0.021	347
rs2955117	0.0175 [−0.3058, 0.3407]	0.915	−0.0038 [−0.3261, 0.3186]	0.982	338
rs12037376	0.0445 [−0.1720, 0.2610]	0.686	0.0087 [−0.2087, 0.2261]	0.937	330
rs9861425	−0.0607 [−0.2840, 0.1627]	0.594	−0.0426 [−0.2659, 0.1806]	0.707	347

Notes. Gestational age at first delivery (weeks) was analyzed using separate linear regression models for each SNP. SNPs were coded additively as 0, 1, or 2 copies of the minor allele. Adjusted models included maternal age at first delivery. β represents the change in gestational age per additional minor allele. Sample size varied because of missing genotype data. Nominal significance was set at *p* < 0.05; the Bonferroni-corrected threshold was *p* < 0.0125. Abbreviations: SNP, single-nucleotide polymorphism; CI, confidence interval.

## Data Availability

The data that support the findings of this study are available from the corresponding author upon reasonable request.

## Supplementary Materials

The following supporting information can be downloaded at: https://www.mdpi.com/article/10.3390/jcm15114269/s1, Table S1: Available SNPs in the Japonica Array dataset and representative SNP selection; Table S2: Post hoc power analysis for age-adjusted additive genetic models; Table S3: Exploratory logistic regression analysis for preterm birth under an additive genetic model; Table S4: Sensitivity analyses using dominant and recessive genetic models.

## Abbreviations

The following abbreviations are used in this manuscript:

GWAS	Genome-wide association study
SNP	Single-nucleotide polymorphism
DNA	Deoxyribonucleic acid
JPT	Japanese in Tokyo
EAS	East Asian
EUR	European
AFR	African
