# Supplementary material for: Maternal Genetic Variants and Gestational Duration: A Replication Study in a Japanese Cohort
Source: J Clin Med. 2026 Jun 1;15(11):4269. doi: 10.3390/jcm15114269 (PMC13257595; doi:10.3390/jcm15114269)
Supplement: Supplementary file 1 [file jcm-15-04269-s001.zip › jcm-4302477-supplementary.pdf]

Supplementary Table S1. Available SNPs in the Japonica Array and representative SNP. selection

Gene/locus	Available SNPs in the Japonica Array dataset	Selected representative SNP	Reason for selection
EBF1	rs2946169, rs2946171	rs2946169	Similar results; clearer allele annotation
EEFSEC	rs200745338, rs2955117	rs2955117	SNV selected over indel for simpler allele coding
WNT4	rs56318008, rs12037376	rs12037376	Similar results; clearer allele coding
ADCY5	rs4383453, rs9861425	rs9861425	Higher allele frequency and better statistical stability

Supplementary Table S2. Post hoc power analysis for age-adjusted additive genetic models.

SNP	N	Observed β	Power at α=0.05	Power at α=0.0125	Minimum detectable β with 80% power at α =0.05	Minimum detectable β with 80% power at α =0.0125
rs2946169	347	−0.2679	0.639	0.426	0.324	0.387
rs2955117	338	−0.0038	0.050	0.013	0.460	0.550
rs12037376	330	0.0087	0.051	0.013	0.310	0.371
rs9861425	347	−0.0426	0.066	0.019	0.319	0.381

Note: Power was estimated using the observed regression coefficient, standard error, and residual degrees of freedom from the age-adjusted additive linear regression model for each SNP. The Bonferroni-corrected threshold was α = 0.0125. Minimum detectable β indicates the absolute per-allele effect size in weeks required to achieve 80% power.

Supplementary Table S3. Exploratory logistic regression analysis for preterm birth under an additive genetic model.

SNP	Odds ratio	95% CI	P value
rs2946169	1.29	0.507-3.30	0.590
rs2955117	0.399	0.0508-3.13	0.382
rs12037376	0.673	0.251-1.80	0.431
rs9861425	0.499	0.165-1.51	0.219

Note: Preterm birth was defined as delivery before 37 completed weeks of gestation. Odds ratios were estimated using separate logistic regression models for each SNP, adjusted for maternal age at first delivery. SNPs were coded as 0, 1, or 2 according to the number of minor alleles and analyzed under an additive genetic model. Because the number of preterm births was small, these results should be interpreted as exploratory.

Supplementary Table S4. Sensitivity analyses using dominant and recessive genetic models.

SNP	Genetic model	Adjusted β [95% CI]	Adjusted p
rs2946169	Dominant	−0.2848 [−0.5883, 0.0186]	0.066
rs2946169	Recessive	−0.5397 [−1.0484, −0.0309]	0.038
rs2955117	Dominant	0.0928 [−0.2587, 0.4443]	0.604
rs2955117	Recessive	−1.2756 [−2.5414, −0.0098]	0.048
rs12037376	Dominant	0.0233 [−0.3389, 0.3855]	0.899
rs12037376	Recessive	0.0008 [−0.3543, 0.3559]	0.997
rs9861425	Dominant	−0.0398 [−0.3565, 0.2770]	0.805
rs9861425	Recessive	−0.0845 [−0.5138, 0.3447]	0.699

Note: Models were adjusted for maternal age at first delivery. β represents the difference in gestational age at first delivery (weeks) compared with the reference genotype group. Dominant models compared minor-allele carriers with major-allele homozygotes, and recessive models compared minor-allele homozygotes with heterozygotes/major-allele homozygotes.
